# Kernel Conversion for Robust Quantitative Measurements of Archived Chest Computed Tomography Using Deep Learning-Based Image-to-Image Translation

**DOI:** 10.3389/frai.2021.769557

**Published:** 2022-01-17

**Authors:** Naoya Tanabe, Shizuo Kaji, Hiroshi Shima, Yusuke Shiraishi, Tomoki Maetani, Tsuyoshi Oguma, Susumu Sato, Toyohiro Hirai

**Affiliations:** ^1^Department of Respiratory Medicine, Graduate School of Medicine, Kyoto University, Kyoto, Japan; ^2^Institute of Mathematics for Industry, Kyushu University, Fukuoka, Japan

**Keywords:** medical imaging, deep learning, lung, computed tomography, chronic obstructive pulmonary disease (COPD), emphysema, reconstruction kernel

## Abstract

Chest computed tomography (CT) is used to screen for lung cancer and evaluate pulmonary and extra-pulmonary abnormalities such as emphysema and coronary artery calcification, particularly in smokers. In real-world practice, lung abnormalities are visually assessed using high-contrast thin-slice images which are generated from raw scan data using sharp reconstruction kernels with the sacrifice of increased image noise. In contrast, accurate CT quantification requires low-contrast thin-slice images with low noise, which are generated using soft reconstruction kernels. However, only sharp-kernel thin-slice images are archived in many medical facilities due to limited data storage space. This study aimed to establish deep neural network (DNN) models to convert sharp-kernel images to soft-kernel-like images with a final goal to reuse historical chest CT images for robust quantitative measurements, particularly in completed previous longitudinal studies. By using pairs of sharp-kernel (input) and soft-kernel (ground-truth) images from 30 patients with chronic obstructive pulmonary disease (COPD), DNN models were trained. Then, the accuracy of kernel conversion based on the established DNN models was evaluated using CT from independent 30 smokers with and without COPD. Consequently, differences in CT values between new images converted from sharp-kernel images using the established DNN models and ground-truth soft-kernel images were comparable with the inter-scans variability derived from repeated phantom scans (6 times), showing that the conversion error was the same level as the measurement error of the CT device. Moreover, the Dice coefficients to quantify the similarity between low attenuation voxels on given images and the ground-truth soft-kernel images were significantly higher on the DNN-converted images than the Gaussian-filtered, median-filtered, and sharp-kernel images (*p* < 0.001). There were good agreements in quantitative measurements of emphysema, intramuscular adipose tissue, and coronary artery calcification between the converted and the ground-truth soft-kernel images. These findings demonstrate the validity of the new DNN model for kernel conversion and the clinical applicability of soft-kernel-like images converted from archived sharp-kernel images in previous clinical studies. The presented method to evaluate the validity of the established DNN model using repeated scans of phantom could be applied to various deep learning-based image conversions for robust quantitative evaluation.

## Introduction

Long-term exposure to cigarette smoke causes damage to the parenchyma and airways in the lungs and lung cancer. Following repeated injuries, smokers may develop emphysema and airway remodeling, leading to chronic obstructive pulmonary disease (COPD) (Vogelmeier et al., 2017). Chest computed tomography (CT) is widely used to screen for lung cancer and simultaneously provides information of emphysema, airway diseases, and even extra-pulmonary abnormalities regarding coronary artery disease, muscle wasting, and bone mineral density loss (Ohara et al., 2008; Mcdonald et al., 2014; Labaki et al., 2017). However, a variability in scanning conditions such as different reconstruction kernels reduces the reproducibility of the quantitative measurements on chest CT (Gierada et al., 2010).

In real-world clinical practice, lung abnormalities are visually assessed by experts on high-contrast thin-slice images that are generated from raw scan data using sharp reconstruction kernels with the sacrifice of increased image noise. In contrast, low-contrast thin-slice images with low noise, which are generated using soft reconstruction kernels, are more appropriate for quantitative measurements. Indeed, increased noise on sharp-kernel images affects quantitative measurement of emphysema on CT (Gierada et al., 2010; Gallardo-Estrella et al., 2016). Nonetheless, only sharp-kernel thin-slice images are archived in many medical facilities due to the limited data storage space. Since the acquisition of CT data imposes radiation exposure and prospective collection of longitudinal CT data requires a long time and cost, computational methods to convert sharp-kernel high-contrast images to soft-kernel-like low-contrast images should be established to reuse archived sharp-kernel CT data and to perform robust quantitative measurements in completed previous studies.

In the field of image processing, deep learning-based techniques have been rapidly updated. Studies have proposed the use of a convolutional neural network to perform kernel conversions to reduce effects of different kernels on quantifying emphysema and extracting radiomics features of nodules and masses on chest CT (Choe et al., 2019; Lee et al., 2019; Bak et al., 2020). In those studies, differences in CT values between converted and ground-truth images were calculated to evaluate the accuracy the image conversion. However, acceptable differences in CT values in terms of clinical utility remain unestablished.

This study aimed to establish and validate a deep learning-based model to convert images to those with different kernels, particularly sharp-to-soft kernel conversion on chest CT. For improved validation process, this study used phantoms that can be repeatedly scanned without concerns for radiation exposure to estimate acceptable differences in CT values for kernel conversion methods. During the careful analysis, it was found that the error profile varies over different regions. Therefore, conversion error was analyzed not only in regions with a broad range of CT values (−1,000 to 1,000 HU) but also in regions with specific range of CT values such as lung parenchyma (−1000 to −500 HU), muscle and fat (−200 to 200 HU), and calcification and bone (>130 HU) (Vieira et al., 1998; Coxson et al., 1999; Alluri et al., 2015; Popuri et al., 2016). Based on the analysis, a novel conversion method to fuse outputs of multiple neural networks was developed. For the validation of clinical applicability, emphysematous change, intramuscular adipose tissue and coronary artery calcification on converted images were compared to those on ground-truth images. For the reproducibility and convenience, the codes and the trained models used in this study were made available online in a ready-to-use form (https://github.com/shizuo-kaji/CTKernelConversion).

## Materials and Methods

### Acquisition of Chest CT Scans of Patients and CT Scans of Phantoms

**Figure 1:**
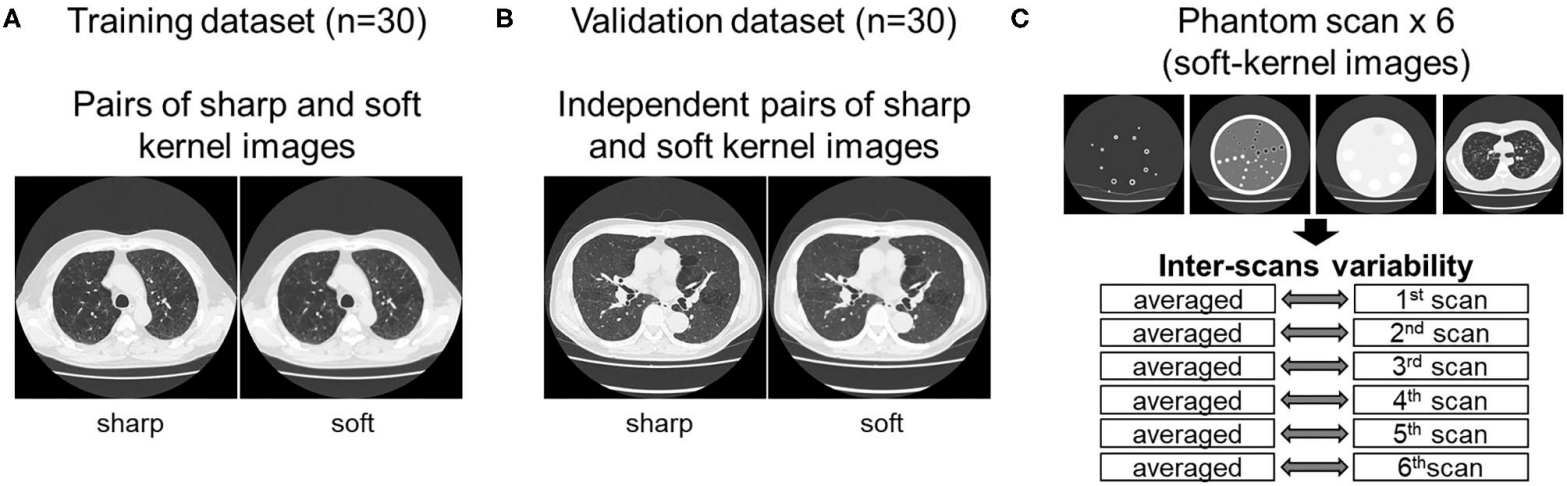
Datasets used to establish and validate image conversion. **(A)** The training dataset comprised pairs of sharp and soft reconstruction kernel images from 30 smokers. The validation of deep learning-based conversion was performed using independent pairs of sharp and soft reconstruction kernel images from 30 smokers [validation dataset **(B)**], and phantom CT that were repeatedly scanned six times **(C)**. Inter-scans variability was assessed by comparing each scan to the averaged CT values from all six scans.

As shown in Figure 1, this study used inspiratory chest CT data in smokers with and without COPD who visited the outpatient COPD clinic in the University Hospital between 2016 and 2020. The data comprising of CT images acquired from 60 patients was divided into 30 training and 30 validation datasets. Lung function was measured using a Chestac-65V (Chest MI Corp., Tokyo, Japan) and a diagnosis of COPD was confirmed based on post-bronchodilator forced expiratory volume in one sec (FEV_1_)/forced vital capacity (FVC) <0.7 and respiratory symptoms. All chest CT scans were obtained at full-inspiration using an Aquilion Prime Scanner (Cannon Medical Systems, Otawara, Japan) that was routinely calibrated with air and water phantoms. The scanning conditions were as follows: 0.5-mm collimation, 500-ms scan time, 120 peak kilovoltage, and auto-exposure control. Raw data were converted to 512 × 512 matrix images with 0.5 and 1.0 mm slice thickness using sharp (FC51) and soft (FC13) reconstruction kernels, respectively. Additionally, we used a set of phantom tubes that mimicked airways, urethan and acrylic foam mimicking lung parenchyma, hydroxyapatite phantom that mimicked the bone mineral, and human body phantom. The phantom was repeatedly scanned six times to evaluate inter-scans variability using the same scanner under the scanning conditions except auto-exposure control. The Ethics Committee of the Kyoto University Hospital approved the study (approval no. R1323). The requirement for informed consent from patients was waived due to the retrospective analysis of the data.

### Development of Image Conversion Method

**Figure 2:**
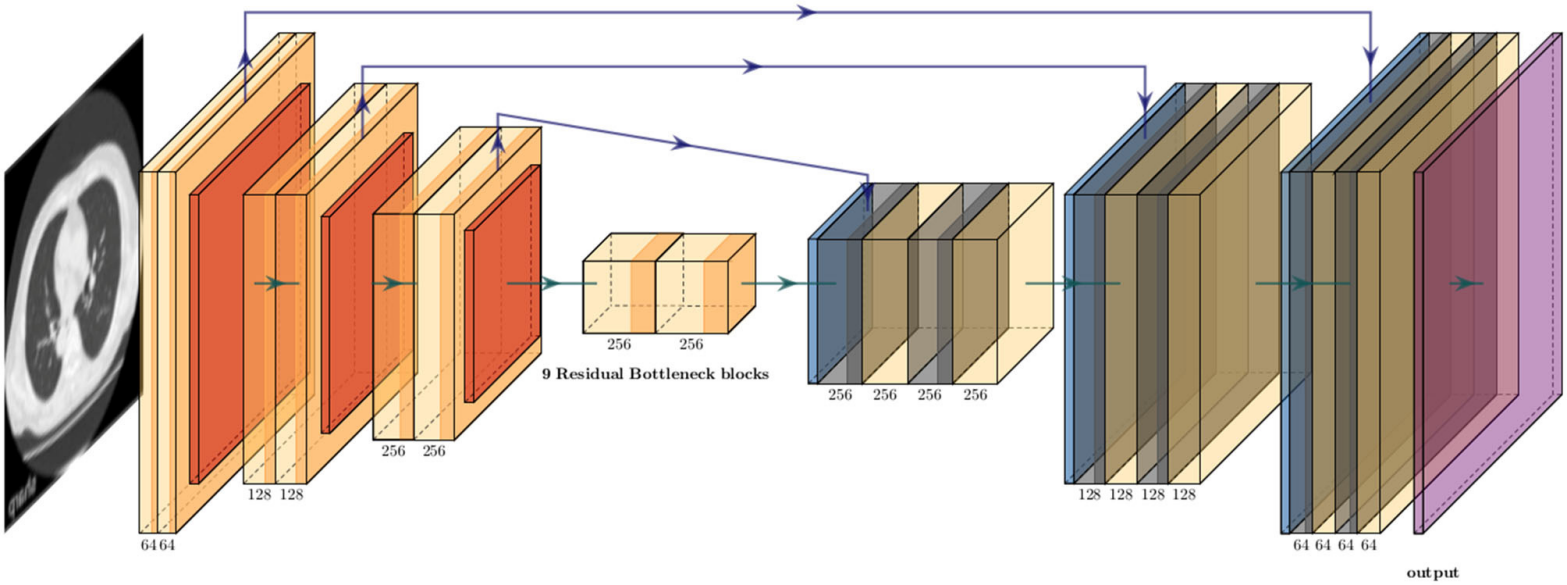
Schematic explanation of deep neural convolutional network in this study. The network consisted of three down-sampling layers followed by nine bottleneck layers of residual blocks flowed by three up-sampling layers. The instance normalization and the ReLU activation function were applied to each layer expect the last. The arctan function was applied as the final activation. Skip connections were formed between the corresponding down and up layers.

**Figure 3:**
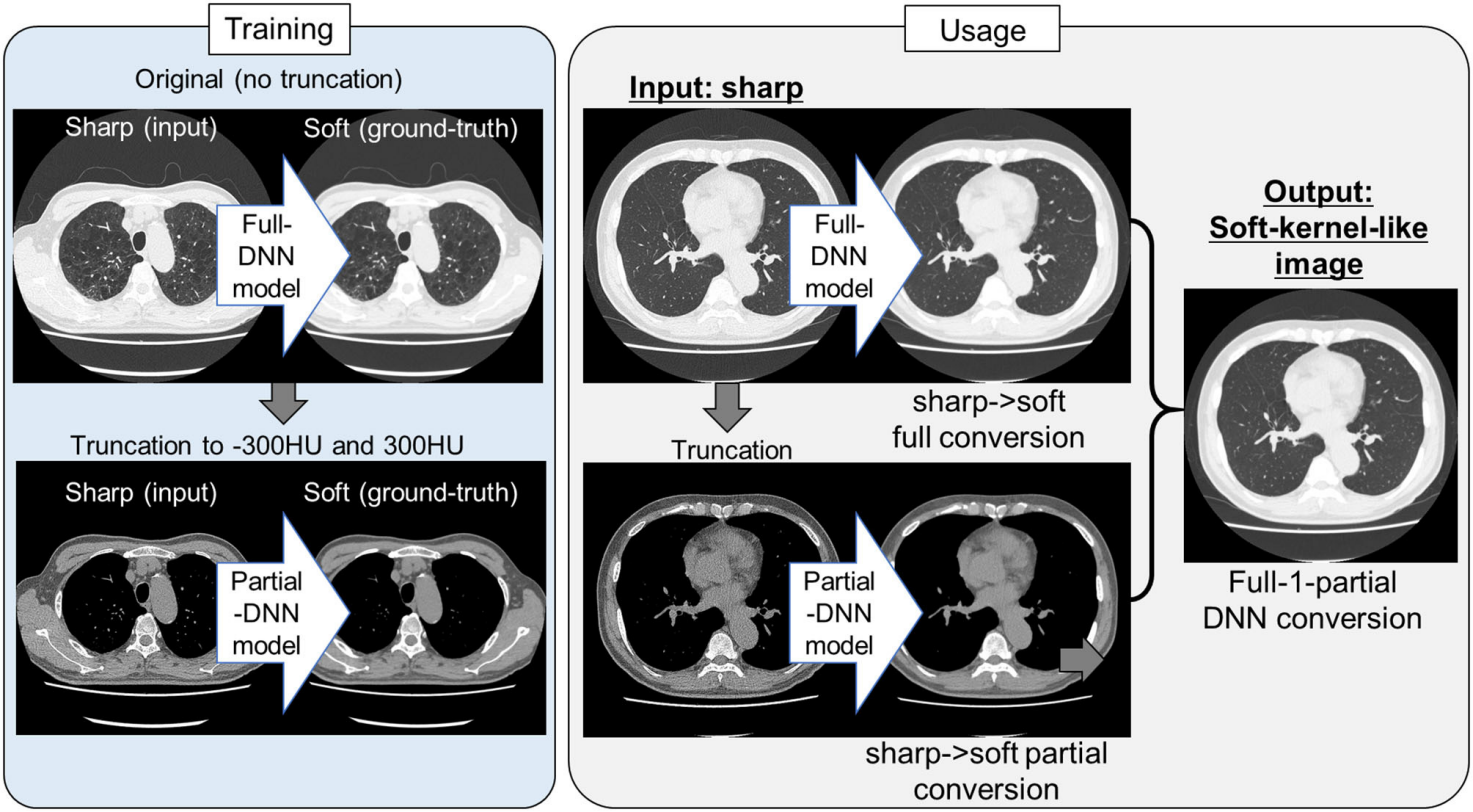
Protocol for the present sharp to soft kernel image conversions using full and partial deep neural networks. In training deep neural network (DNN), pairs of original sharp kernel and soft kernel images were used as input and ground-truth, respectively. One model fully used these pairs without truncation (Full DNN model). The other model partially used these pairs by truncating CT values to −300 and 300 HU (Partial DNN model). By using the full-DNN model and partial-DNN model, sharp-kernel images in an independent validation dataset, were converted to soft-kernel-like images, respectively. Then converted two images were merged to finalize soft-kernel-like images (Full-1-parital DNN conversion).

Because the training CT datasets (*n* = 30) comprised 0.5-mm-thickness images reconstructed with sharp-kernel (sharp-kernel image) and 1.0-mm-thickness images reconstructed with soft-kernel (soft-kernel image), every other sharp-kernel image was selected to match the slice location to that corresponding soft-kernel image. Then, ~300 to 400 pairs of sharp-kernel and soft-kernel images were prepared for each of the 30 patients, and total 11,052 pairs were used to train a deep convolutional neural network (DNN) to convert sharp-kernel images to soft-kernel images (or convert soft-kernel images to sharp-kernel images). DNN was based on U-Net (Ronneberger et al., 2015) and implemented in Python by Kaji and Kida (2019) (https://github.com/shizuo-kaji/PairedImageTranslation). As shown in Figure 2, the network takes sharp-kernel images as input and outputs soft-kernel-like images. The network was trained to minimize the combination of the absolute and the squared errors between the soft-kernel-like images and the corresponding ground-truth soft-kernel images. It took 20 h on a personal computer with a single Nvidia 2080 Ti to train the network for 40 epochs. Several techniques such as learning rate scheduling are used to stabilize the training. The reader is referred to the code for the details. The novelty in the network design peculiar to this study lies in the *region-wise learning* as explained below. In the error analysis presented in a later section, it was discovered that the accuracy of the conversion deteriorated for the region with CT values from −200 to 200 HU. To circumvent this problem, an extra network (the *partial-DNN model*) was trained for the conversion from the sharp-kernel to the soft-kernel images both truncated to the range of −300 to 300 HU (Figure 3) in addition to the *full-DNN model* that was trained with non-truncated images. The final converted image *I*_*final*_ was generated by fusing the outputs of the two models by a weighted sum:


$$\begin{array}{l} I_{final}\left(x\right)=w\;I_{partial}\left(x\right)+\left(1-w\right)I_{full}\left(x\right),\\ \;w=\max\left(\min\left(\max\left(1-\frac{I_{partial}\left(x\right)+230}{60},\frac{I_{partial}\left(x\right)-170}{60}\;\right),1\right),0\;\right), \end{array}$$

where *I*_*partial*_(*x*) (*I*_*full*_(*x*), respectively) is the CT value of the output of the partial-DNN model (that of the full-DNN model, respectively) located at a voxel *x*.

### Validation of Established Conversion Method

Two tests were performed to examine the validity of conversion methods.

#### Differences in CT Values Between Newly Converted Images and Ground-Truth Images

When sharp-kernel images were converted to new images (converted sharp-kernel images), soft-kernel images were considered ground-truth and the difference was measured. Because regions with different CT values might affect the performance of the conversions, the differences in CT values between new images and ground-truth images were evaluated not only in the entire regions but also in local regions limited by CT values such as regions with CT values between −1,000 and −600 HU mainly reflecting lung parenchyma, those between −600 and −200 HU, those between −200 and 200 HU mainly reflecting blood vessel, muscle, and adipose tissue, those between 200 and 600 HU mainly reflecting coronary artery calcification and bone mineral, and those between 600 and 1000 HU. Additionally, CT values of repeated phantom CT scans (*n* = 6) were averaged and the differences between CT values on each scan and the averaged CT values were calculated to evaluate a variation in CT values that a CT scanner intrinsically generates. The distributions of the differences in CT values between the converted images and the ground-truth images were compared to those in CT values calculated from repeated phantom scans. The variation of repeated phantom scans indicates the intrinsic noise due to the measurement limitation, and conversion error below this level is deemed to be inevitable and acceptable. Similarly, the conversion in the opposite direction was also performed and evaluated in which soft-kernel images were converted to sharp-kernel-like images, and the sharp-kernel images were considered as ground-truth.

#### Comparisons of Quantification of Clinically Relevant CT Findings Between Newly Converted Images and Ground-Truth Images

The validity of conversion method was confirmed from clinical perspectives. Emphysema was radiographically identified as voxels with CT values <-950 HU. The percentage of these low attenuation voxels to those in the entire lungs (LAV%) was a standard CT index for emphysema severity. Additionally, the sharp kernel images were converted using median and Gaussian filters, respectively. LAV% was calculated on soft-kernel and sharp-kernel images as well as DNN-based converted images and median and Gaussian filtered images as previously reported (Tanabe et al., 2021c). The quality of emphysema segmentation was evaluated using the Dice coefficients (Rao, 1948), which quantified the similarity between low attenuation voxels on given images and the ground-truth soft-kernel images.

Moreover, because comparisons of LAV% between images with different reconstructions evaluate the validity of conversion method for regions with lower CT values, this study also evaluated intramuscular adipose tissue (IMAT) in the pectoralis major and minor muscles on the anterior chest. Pectoralis major and minor muscles were manually segmented on an axial slice above the aortic arch (Mcdonald et al., 2014; Diaz et al., 2018; Bak et al., 2019). Adipose tissue was defined as regions <-30 HU (Popuri et al., 2016; Donovan et al., 2021) and the percentage of IMAT to the total area of pectoralis major and minor muscles (IMAT%) was calculated. Moreover, regions including coronary arteries were manually traced and coronary artery calcium (CAC) that was defined as regions with >130 HU were identified. CAC volume was calculated by multiplying the total number of CAC voxel by volume of each voxel (Alluri et al., 2015).

### Statistics

Statistical analyses were performed using R version 3.5.1 [R Foundation for Statistical Computing (R Core Team, 2021)]. Data were expressed as median and 1st and 3rd quantiles. A *p* < 0.05 was considered statistically significant. The Dice coefficients to quantify the similarity between low attenuation voxels on given images and the ground-truth soft-kernel images were compared between the DNN-converted images, the Gaussian-filtered, median-filtered, and sharp-kernel images using multiple paired *t*-tests with Bonferroni correction. The inter-methods variability in LAV%, IMAT%, and CAC volume was compared using Bland-Altman plots, in which the average values were plotted on the x-axis and the difference values were plotted on the y-axis (Bland and Altman, 1986, 1996).

## Results

### Characteristics of Study Subjects

Table 1 shows demographics of smokers with and without COPD. The training dataset comprised 30 smokers with COPD whereas the validation dataset comprised 25 smokers with COPD and 5 smokers without COPD.

**Table 1:** Clinical information in two datasets.

	**Training data (*n* = 30)**	**Validation data (*n* = 30)**
Age, years	72.4 ± 6.9	70.3 ± 8.9
Male/Female	27/3	24/6
Height, cm	164.0 ± 0.6	165.0 ± 0.6
Body mass index	22.3 ± 3.3	22.5 ± 3.3
Current/former smoker	6/24	10/20
Smoking pack-years	67.7 ± 39.2	48.1 ± 22.6
FEV_1_, % predicted	67.6± 19.3	71.4 ± 18.6
FVC, % predicted	91.7 ± 18.1	90.9 ± 14.8
FEV_1_/FVC	0.56 ± 0.09	0.61 ± 0.14

*FEV_1_, forced expiratory volume in 1 s; FVC, forced vital capacity*.

### DNN-Based Conversion Model

**Figure 4:**
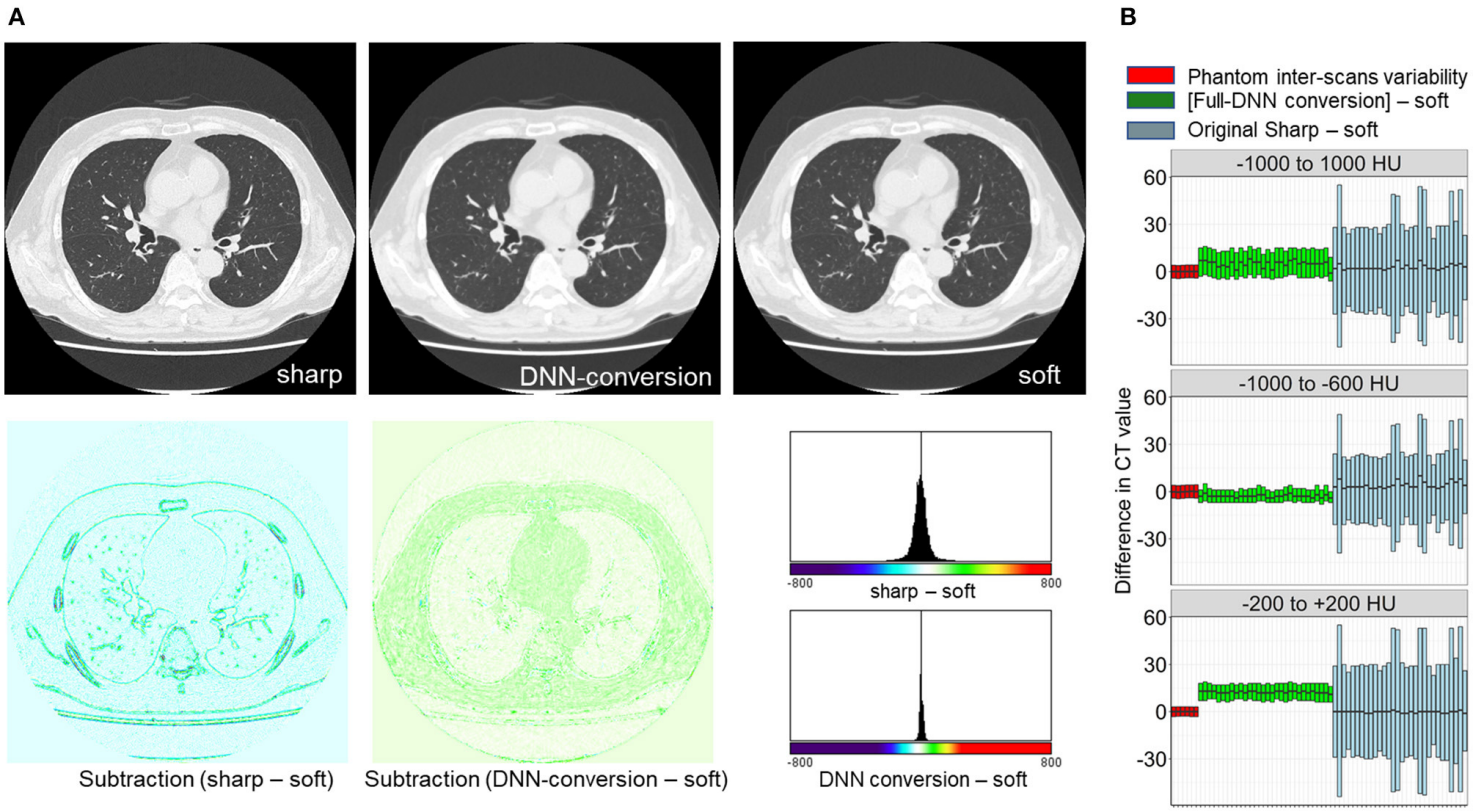
Differences in CT values between deep learning-based (sharp to soft) kernel-converted images and ground-truth soft kernel images. **(A)** Sharp kernel images in the validation dataset (*n* = 30) were converted using a deep neural network without truncating input images (FULL-DNN conversion). The difference in CT values between converted and soft kernel images was smaller than the differences in CT values between the original sharp and soft kernel images. **(B)** These differences in CT values were assessed in regions with CT values between −1,000 and 1,000 HU (broad range), those between −1,000 and −600 HU, and those between −200 and 200 HU. In regions with CT values between −200 and 200 HU, the differences in CT values between converted and soft kernels were higher than differences between averaged phantom CT and each phantom CT.

First, the full-DNN model was trained using paired sharp-kernel images (input) and soft-kernel images (ground-truth) to convert sharp-kernel images to new images whose values were as close to the ground-truth soft-kernel images as possible for the whole range of CT values. Based on the full-DNN model, sharp-kernel images in the validation dataset were converted. Figure 4A shows that differences in CT values between the full-DNN-based converted and soft-kernel images were smaller than those between the sharp-kernel and soft-kernel images. Figure 4B shows distributions of differences in CT values for regions with CT values between −1,000 and 1,000 HU that covered the entire thoracic cage, those between −1,000 and −600 HU that mainly covered the lung parenchyma, and those between −200 and 200 HU that mainly covered the vessel, muscle, and adipose tissues. The difference between the converted images and soft-kernel images in regions between −1,000 and −600 HU was as small as that among the repeated phantom scans. However, the median values were higher in the converted images than in the soft-kernel images for the regions between −1,000 to 1,000 HU and −200 to 200 HU, suggesting that the full-DNN-based conversion of sharp-kernel to soft-kernel-like images was good in regions between −1,000 and −600 HU, but not in regions between −200 and 200 HU.

**Figure 5:**
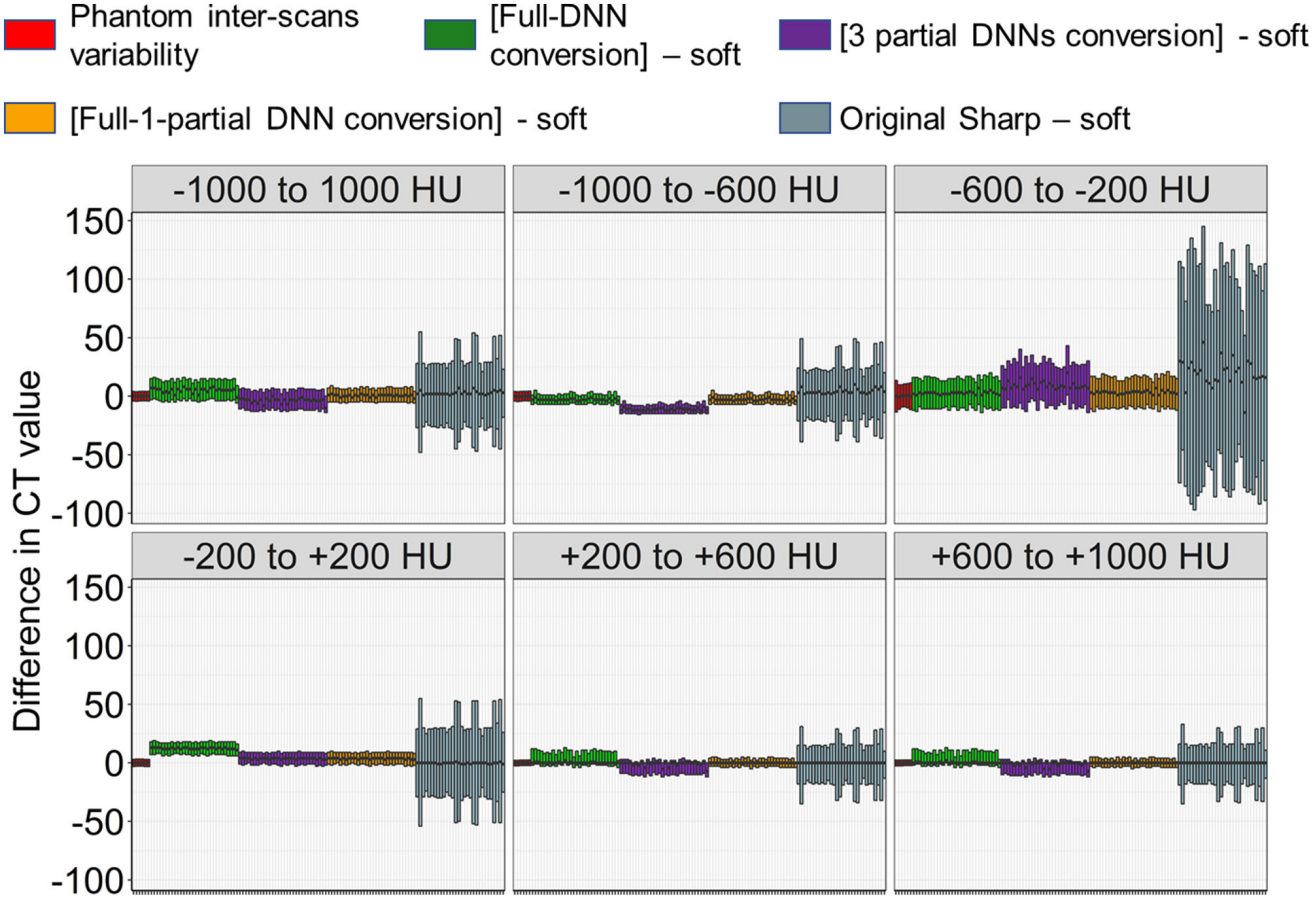
Differences in CT values between images converted using a combination of full and partial deep neural network models and the ground-truth soft kernel images. An inter-scans variability was estimated as differences between the averaged CT values of six repeated scans of the phantom and each of them. Deep neural network (DNN)-based conversion from sharp kernel images in the validation dataset (*n* = 30) were performed without truncating CT images for training (Full-DNN conversion) and with truncating the CT values to a given range such as −300 to 300 HU (partial DNN). Full-1-partial DNN conversion indicates a combination of images converted with the full DNN and images converted with a partial DNN with truncation of CT values to −300 to 300 HU. Additionally, 3 partial DNN conversions were performed by combining partial DNN with truncation of CT values to −2,048 to 0, −300 to 300, and 0 to 1,500 HU. The differences in CT values between each converted images and ground-truth soft-kernel images were compared to the phantom-derived inter-scans variability.

Next, as shown in Figures 3, 5, the partial-DNN model was trained by truncating CT values to −300 and 300 HU on sharp-kernel and soft-kernel images to improve the quality of conversion in regions between −200 and 200 HU. Then, sharp-kernel images in the validation dataset were converted by the partial-DNN model and the full-DNN-based model, which were fused to full-1-partial-DNN converted images. Furthermore, two other DNN models using images whose CT values were truncated to −2,048 to 0 HU and to 0 to 1,500 HU were made. Images generated based on the 3 partial-DNN models (−2,048 to 0, −300 to 300 HU, and 0 to 1,500 HU) were fused to the 3-partial-DNN converted images. Figure 5 shows that the error distribution of the full-1-partial-DNN converted images was closer to the inter-scans variability of CT values on phantom CT, compared to that of the full-DNN converted images, the 3-partial-DNN converted images, and sharp-kernel images in all CT value regions.

### Validation of Quantitating Emphysema, Intramuscular Adipose Tissue, and Coronary Artery Calcification Using Converted Images

**Figure 6:**
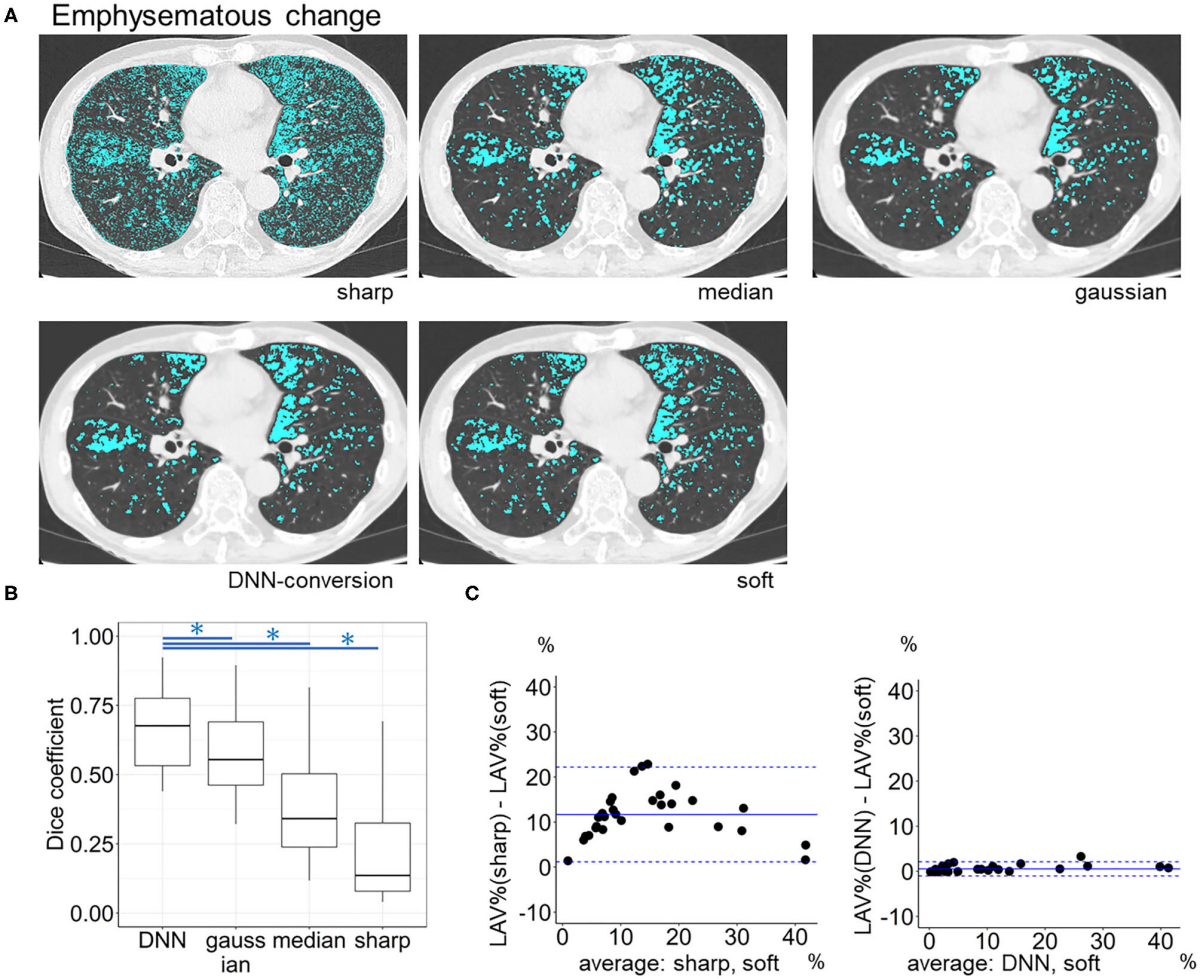
Emphysema quantification on deep learning-based kernel-converted images. **(A)** Emphysematous change (blue) on the original sharp-kernel, median-filtered, Gaussian-filtered (sharp to soft), kernel conversion, and original soft-kernel images. **(B)** The Dice coefficients quantified the similarity between low attenuation voxels on given images and the ground-truth soft-kernel images in the validation dataset (*n* = 30). *indicates *p* < 0.001 based on paired *t*-tests with Bonferroni correction. **(C)** Bland-Altman plots of the extent of emphysematous change, assessed as low attenuation volume % (LAV%), show that LAV% on converted and soft-kernel images were close to each other. Solid blue line indicates the mean difference (bias) between the two measurements. Upper and lower dashed lines indicate 95% limits of agreement.

**Figure 7:**
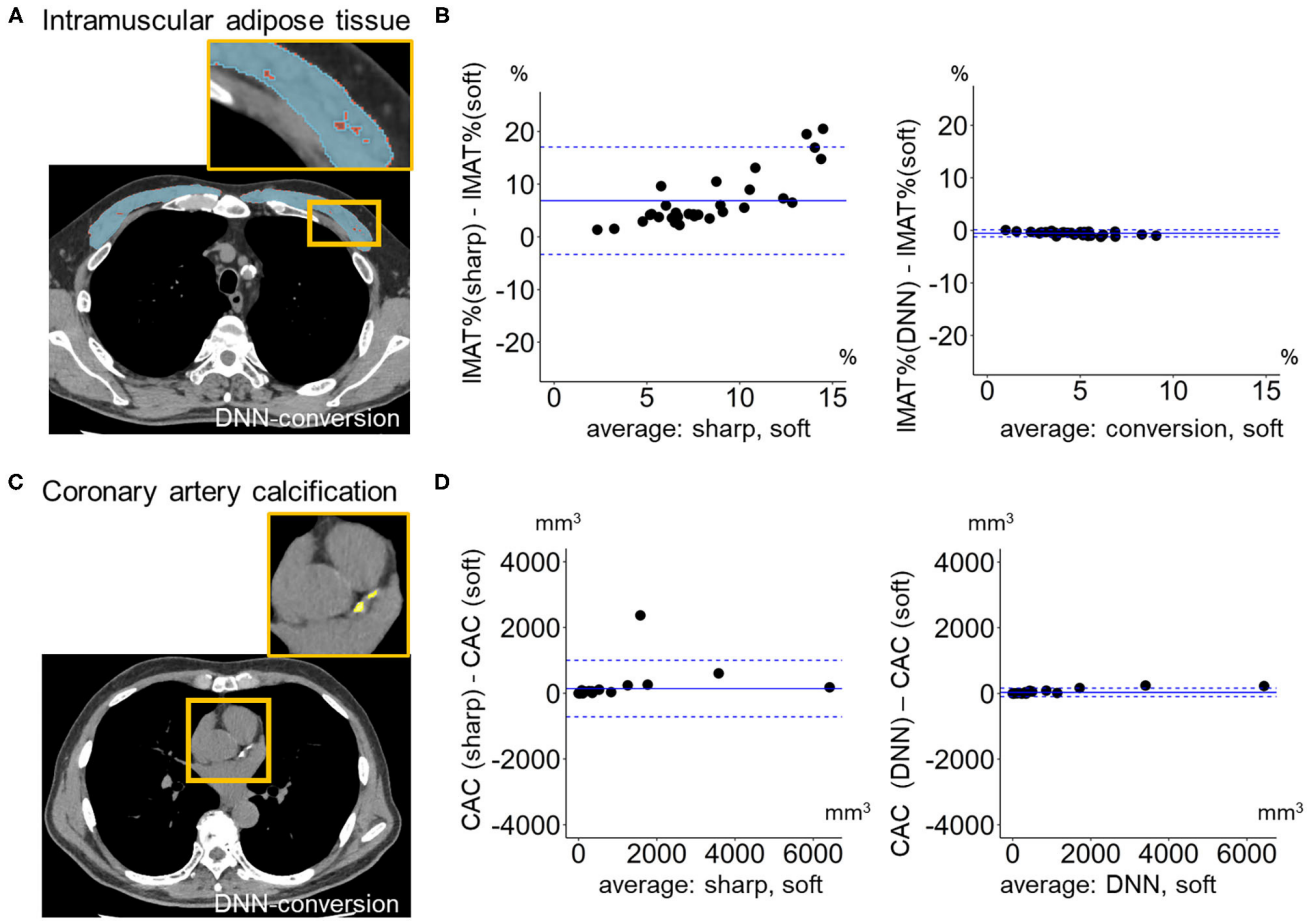
Quantification of intramuscular adipose tissue and coronary artery calcification on deep learning-based kernel-converted images. **(A)** Intramuscular adipose tissue (IMAT, yellow) in pectoral muscles (blue) on the DNN-converted image near the top of the aortic arch. **(B)** Bland-Altman plots of the volume percentage of IMAT to pectorals muscles (IMAT%) showed that IMAT% on DNN-converted and soft-kernel images in the validation dataset (*n* = 30) were close to each other. **(C)** Coronary artery calcification (CAC, yellow) on DNN-converted image. **(D)** Bland-Altman plots of the volume of CAC showed that CAC volume on DNN-converted and soft-kernel images was close to each other. Solid blue line indicates the mean difference (bias) between the two measurements. Upper and lower dashed lines indicate 95% limits of agreement.

Figure 6 compares LAV% on the full-1-partial-DNN-converted images, sharp-kernel images, median-filtered images, Gaussian-filtered images, and soft-kernel (ground-truth) images. The Dice coefficients for low attenuation voxels using the soft-kernel images as the reference were significantly higher on the DNN-converted images than the Gaussian-filtered, median-filtered, and sharp-kernel images (*p* < 0.001 based on paired-t tests with Bonferroni correction). Moreover, Bland-Altman plot shows that the difference between the converted and soft-kernel images was much smaller than that between the sharp-kernel and soft-kernel images [Bias (95% limits of agreement), 0.54 (−1.04, 2.12) vs. 11.68 (1.16, 22.20)%, respectively]. Figure 7 shows that IMAT% of the pectoralis major and minor muscles and CAC volume in the converted and soft-kernel images were close to each other [Bias (95% limits of agreement) for IMAT% and CAC volume = −0.56 (−1.23, 0.12)% and 31.67 (−98.40, 161.74) mm^3^].

#### Conversion of Soft-Kernel Images to Sharp-Kernel-Like Images

**Figure 8:**
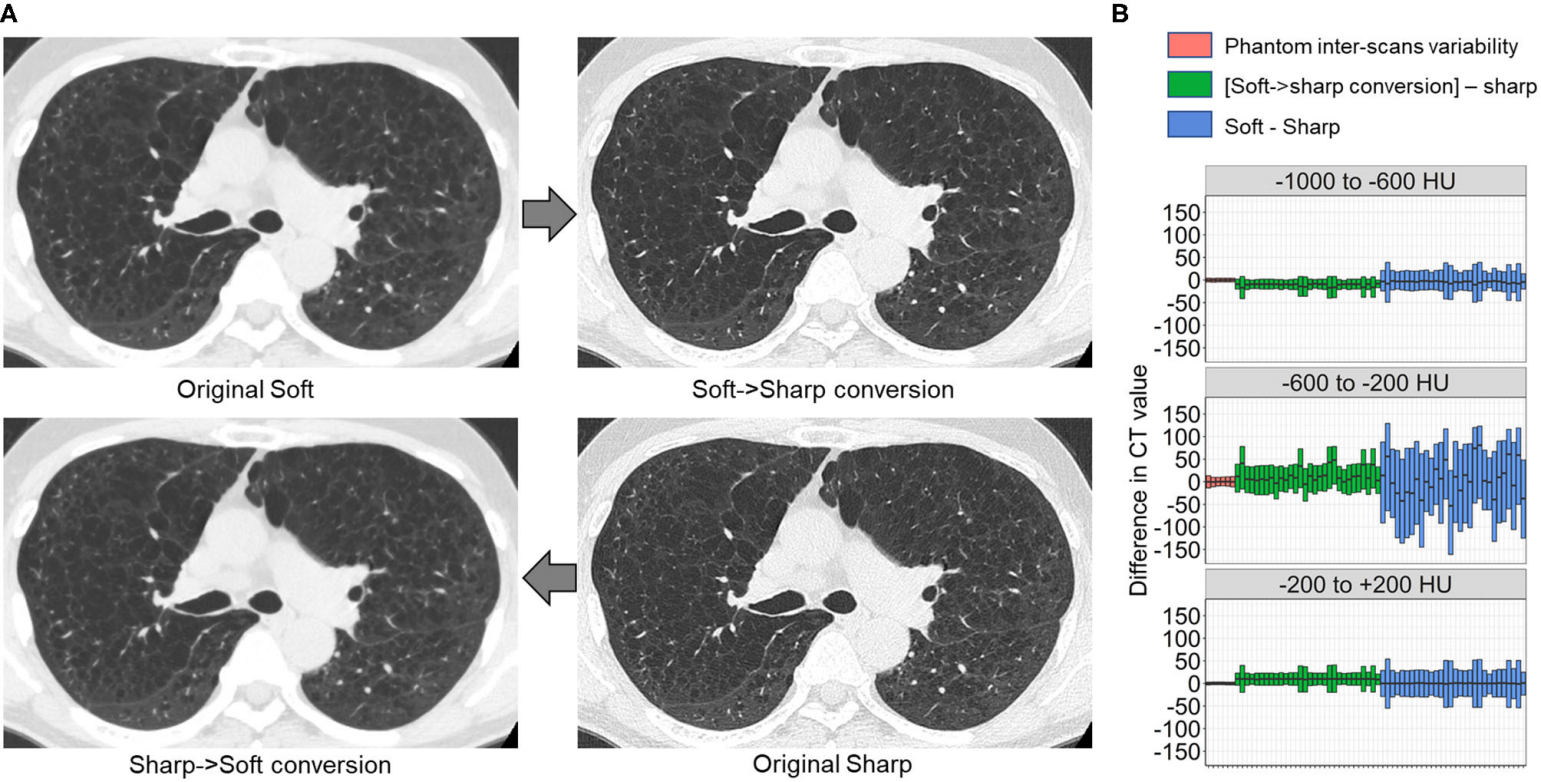
Differences in CT values between deep learning-based kernel-converted (soft to sharp) images and ground-truth sharp-kernel images. **(A)** Deep learning-based soft-to-sharp conversion of original soft-kernel images to sharp-kernel-like images. Two deep leaning convolutional networks with and without truncation of CT values to −300 to 300 HU (partial DNN and full DNN) were trained using pairs of soft-kernel images (input) and sharp-kernel-images (ground-truth), and output images from the partial DNN and full DNN were merged based on the same principle as sharp-to-soft conversion method. **(B)** Differences in CT values between converted images and ground-truth sharp images were compared to an inter-scans variability calculated from repeated phantom scans.

Finally, we tested whether the principle for establishing the DNN model to convert sharp-kernel images to soft-kernel-like images was applied to establish the DNN model to convert soft-kernel images to sharp-kernel-like images. The full-DNN model and partial-DNN model were trained using paired soft-kernel images (input) and sharp-kernel images (ground-truth) without truncation and with truncation of CT values to −300 to 300 HU. Based on these two models, the soft-kernel images in the validation dataset were converted to two types of images and fused to sharp-kernel-like images. Figure 8 shows that the error distribution of the full-1-partial-DNN converted images were close to the inter-scans variability of CT values on phantom CT for not only regions with CT values between −1,000 and 1,000 HU but also those with CT values in any specific ranges such as between −1,000 and −600 HU.

## Discussion

This study established and validated the DNN model for kernel conversion of chest CT by comparing differences in CT values between the converted and ground-truth images to those calculated from repeated phantom scans representing the inter-scans variability that a scanner had intrinsically. Moreover, the clinical validity of the kernel conversion was confirmed by showing that quantitative measurements of the well-established CT indices, LAV%, IMAT%, and CAC volume on the converted and ground-truth images were matched well. Therefore, the deep learning-based kernel conversion method presented here contributes to effective reuse of historical chest CT images for robust quantitative measurements, particularly when detailed clinical data and sharp-kernel thin-slice images, but not soft-kernel thin-slice images, were preserved in completed previous longitudinal studies.

One of the main findings of the current study is that neural-network-based CT conversion has varying error profiles with respect to the CT value range. Natural images, with which the foundation of neural-network-based image conversion methods have been developed, have much narrower dynamic range (typically, 8 bit) compared to CT images (typically, 16 bit). Since different CT value ranges correspond to different anatomical structures, CT images encompass a rich structure that makes it a difficult task for a single neural network to deal with. In fact, our careful analysis showed that the accuracy of the conversion deteriorated for the middle CT value region from −200 to 200 HU. A possible explanation is that the sigmoid-type activation function used in the last output layer has a larger gradient in the middle range value, which would lead to poor convergence and less stability in learning the conversion for the CT range around 0 HU. To circumvent this problem, training an additional neural network for the specific range from −300 to 300 HU and fusing the output of multiple networks was proposed in this study. The resulting conversion was validated with both the CT values and some major clinical indices. Moreover, finding that the combination of 3 partial-DNN models did not improve the conversion error compared to the combination of the two DNN models (a full DNN and a partial DNN) suggests that two networks were sufficient to obtain acceptable soft-kernel-like images. An alternative solution could be the use of a wider and deeper neural network that has a larger capacity. However, larger models are prone to overfitting and poor convergence, particularly when trained with the same amount of data.

Recent technical advances in the field of image processing have invented convolutional neural networks that improved image quality and segmentation of specific regions of medical imaging including chest CT (Kim et al., 2018; Choe et al., 2019; Lee et al., 2019; Bak et al., 2020; Handa et al., 2021; Tanabe et al., 2021a). Indeed, Lee et al. (2019) invented a deep learning-based method to convert CT images into those of different reconstruction kernels and to achieve a more rigorous measurement of emphysema. Bak et al. (2020) also established a deep learning-based method to convert sharp-kernel low-dose CT images to reduce image noises and quantify emphysema more reproducibly. Furthermore, Choe et al. (2019) performed the deep learning-based kernel conversion and succeeded in reducing a variability in radiomics features of pulmonary nodules and masses between different reconstruction kernels. Those previous findings were extended by the present data that proposed the novel method to evaluate the accuracy of the conversion using repeated scans of phantom.

Repeated phantom scans allowed using the difference between CT values from a given scan and those calculated as the average as an index of inter-scans variability. It was found that the differences in CT values between the images converted from sharp-kernel images and the ground-truth soft-kernel images were considered the acceptable level that was compatible with the inter-scans variability. Moreover, the comparisons of the differences in CT values to the phantom-derived inter-scans variability were performed not only in the entire regions defined as pixels with CT values between −1,000 and 1,000 HU but also specific regions defined as pixels with CT values within specific regions such as −1000 to −600 HU. This strategy made it possible to find that when sharp-kernel images were converted based on the full-DNN method only, the distribution of CT values was higher in the converted images than in the ground-truth soft-kernel images.

Because the process of deep learning-based methods such as DNN is hard to interpret like “black-box,” establishing rigorous methods to check the accuracy of image conversions is essential. In this context, the proposed validation process is important for secure use of deep learning-based image conversion in clinical practice, where the top priority is placed on the safety problem.

The Bland-Altman plots showed that LAV%, IMAT%, and CAC volume on the converted sharp-kernel images were compatible with those on the ground-truth soft-kernel images with acceptable levels. Additionally, although the normalization of the reconstruction kernel using filtering may allow accurate quantification of emphysema (Gallardo-Estrella et al., 2016), the present data showed that the Dice coefficient for emphysematous changes on the DNN-based converted images was higher than the Gaussian-filtered and median-filtered images. This finding suggests that the DNN conversion is more appropriate than other filtering methods to quantify emphysema.

It is well-known that the extent of emphysema on CT is associated with various clinical outcomes such as rapid lung function decline, exacerbations, and increased mortality (Haruna et al., 2010; Han et al., 2011; Vestbo et al., 2011; Nishimura et al., 2012). Moreover, CT indices regarding extra-pulmonary comorbidities including sarcopenia and cardiovascular diseases also affect outcomes in patients with COPD (Mcdonald et al., 2014; Tanimura et al., 2016; Bak et al., 2019; Tanabe et al., 2021b). Indeed, increased IMAT and coronary artery calcification have been shown to be associated with poor outcomes in smokers with and without COPD (Williams et al., 2014; Pishgar et al., 2021). Therefore, the found validity of measurement of LAV%, IMAT%, and CAC volume using the DNN-based converted images would help re-analyze previously archived sharp-kernel images to explore better clinical management. Furthermore, since LAV%, IMAT%, and CAC volume reflect low, middle, and high CT values, reproducible measurements of these indices suggest that other CT abnormalities can be reliably quantified using the converted soft-kernel-like images.

The deep learning-based conversion from soft kernel images to sharp-kernel-like images was also established in this study. The differences in CT values between the converted images and the ground-truth sharp kernel images were consistent with those in CT values obtained from repeated phantom scans. Although the conversion from soft to sharp kernel images is less required than the conversion from sharp to soft kernel images, the found accuracy of the soft-to-sharp kernel conversion suggests that the proposed pipeline to establish kernel conversion methods can be applied to various kernels conversion when CT pairs for model training are available.

Dual-energy CT scanners are rapidly emerging worldwide. Because a single contrast-enhanced acquisition can provide both contrast-enhanced images and virtual unenhanced (VUE) images and reduce radiation dose by eliminating the need for the true unenhanced scans, this technique has been used in many fields including vascular imaging (Otrakji et al., 2016). Indeed, VUE images derived from iodine contrast-enhanced CT data can be used to evaluate coronary artery calcification with equal quality to the true unenhanced scans (Yamada et al., 2014). Although VUE images have been used for density analysis such as quantitative emphysema assessment (Lee et al., 2012), we believe that the present image conversion method is applicable to improve the quality of VUE images further and to achieve more robust density-based quantitative measurements in various diseases.

This study has some limitations. First, this study evaluated two reconstruction kernels (sharp kernel = FC51 and soft kernel = FC13) for a single CT scanner. Establishing a new DNN model might be necessary to perform accurate kernel conversion for other kernels and types of scanners. However, this task is not hard because the present method can be applied directly once the dataset is prepared. Furthermore, transfer learning should save the amount of data needed. Second, although the validity of the converted images was confirmed in terms of CT values and clinical CT indices such as LAV%, IMAT%, and CAC volume, clinical impacts of quantitative analysis of the converted images cannot be evaluated due to the small sample size. Third, the present kernel conversion method was not compared to other machine learning-based conversion methods. This should be performed in future studies.

In conclusion, this study established the DNN model for kernel conversion and the method to validate the established model using repeated phantom scan data. The accuracy of the kernel conversion was confirmed by comparing error distributions of CT values on converted images to the phantom-derived inter-scans variability and by showing good agreements in the extents of emphysema, intramuscular adipose tissue, and coronary artery calcification between converted and ground-truth images. The data indicate the clinical applicability of sharp-to-soft kernel conversions to perform robust quantitative CT analyses in regular practice where only sharp-kernel thin-slice images were stored. The presented process of establishing and validating the DNN model using pairs of input and ground-truth images as well as repeated scans of phantom can be applied to various deep learning-based image-to-image translation.

## Data Availability Statement

The raw data supporting the conclusions of this article will be made available by the authors, without undue reservation.

## Ethics Statement

The studies involving human participants were reviewed and approved by the Ethics Committee of the Kyoto University Hospital. Written informed consent for participation was not required for this study in accordance with the national legislation and the institutional requirements.

## Author Contributions

NT, SK, HS, YS, TM, TO, SS, and TH contributed to collecting, analyzing, and interpreting the data. SK developed scripts for deep learning-based kernel conversion. NT, HS, YS, TM, and TO developed scripts for quantitative measurements of CT abnormalities. NT and SK contributed to conceiving and designing the study and performed statistical analysis. NT, SK, HS, YS, TO, SS, and TH contributed to writing the manuscript. All authors contributed to the article and approved the submitted version.

## Funding

This work was partially supported by the Japan Society for the Promotion of Science (JSPS; Grants-in-Aid for scientific research 19K08624).

## Conflict of Interest

The authors declare that the research was conducted in the absence of any commercial or financial relationships that could be construed as a potential conflict of interest.

## Publisher's Note

All claims expressed in this article are solely those of the authors and do not necessarily represent those of their affiliated organizations, or those of the publisher, the editors and the reviewers. Any product that may be evaluated in this article, or claim that may be made by its manufacturer, is not guaranteed or endorsed by the publisher.

## References

[B1] Alluri K. Joshi P. H. Henry T. S. Blumenthal R. S. Nasir K. Blaha M. J. (2015). Scoring of coronary artery calcium scans: history, assumptions, current limitations, and future directions. Atherosclerosis 239, 109–117. 10.1016/j.atherosclerosis.2014.12.040 25585030

[B2] Bak S. H. Kim J. H. Jin H. Kwon S. O. Kim B. Cha Y. K.. (2020). Emphysema quantification using low-dose computed tomography with deep learning-based kernel conversion comparison. Eur. Radiol. 30, 6779–6787. 10.1007/s00330-020-07020-3 32601950

[B3] Bak S. H. Kwon S. O. Han S. S. Kim W. J. (2019). Computed tomography-derived area and density of pectoralis muscle associated disease severity and longitudinal changes in chronic obstructive pulmonary disease: a case control study. Respir. Res. 20:226. 10.1186/s12931-019-1191-y 31638996 PMC6805427

[B4] Bland J. M. Altman D. G. (1986). Statistical methods for assessing agreement between two methods of clinical measurement. Lancet 1, 307–310. 10.1016/S0140-6736(86)90837-8 2868172

[B5] Bland J. M. Altman D. G. (1996). Measurement error and correlation coefficients. BMJ 313, 41–42. 10.1136/bmj.313.7048.41 8664775 PMC2351452

[B6] Choe J. Lee S. M. Do K. H. Lee G. Lee J. G. Lee S. M.. (2019). Deep learning-based image conversion of CT reconstruction kernels improves radiomics reproducibility for pulmonary nodules or masses. Radiology 292, 365–373. 10.1148/radiol.2019181960 31210613

[B7] Coxson H. O. Rogers R. M. Whittall K. P. D'yachkova Y. Pare P. D. Sciurba F. C.. (1999). A quantification of the lung surface area in emphysema using computed tomography. Am. J. Respir. Crit. Care Med. 159, 851–856. 10.1164/ajrccm.159.3.9805067 10051262

[B8] Diaz A. A. Martinez C. H. Harmouche R. Young T. P. Mcdonald M. L. Ross J. C.. (2018). Pectoralis muscle area and mortality in smokers without airflow obstruction. Respir. Res. 19:62. 10.1186/s12931-018-0771-6 29636050 PMC5894181

[B9] Donovan A. A. Johnston G. Moore M. Jensen D. Benedetti A. Coxson H. O.. (2021). Diaphragm morphology assessed by computed tomography in chronic obstructive pulmonary disease. Ann. Am. Thorac. Soc. 18, 955–962. 10.1513/AnnalsATS.202007-865OC 33321048

[B10] Gallardo-Estrella L. Lynch D. A. Prokop M. Stinson D. Zach J. Judy P. F.. (2016). Normalizing computed tomography data reconstructed with different filter kernels: effect on emphysema quantification. Eur. Radiol. 26, 478–486. 10.1007/s00330-015-3824-y 26002132 PMC4712239

[B11] Gierada D. S. Bierhals A. J. Choong C. K. Bartel S. T. Ritter J. H. Das N. A.. (2010). Effects of CT section thickness and reconstruction kernel on emphysema quantification relationship to the magnitude of the CT emphysema index. Acad. Radiol. 17, 146–156. 10.1016/j.acra.2009.08.007 19931472 PMC2818169

[B12] Han M. K. Kazerooni E. A. Lynch D. A. Liu L. X. Murray S. Curtis J. L.. (2011). Chronic obstructive pulmonary disease exacerbations in the COPDGene study: associated radiologic phenotypes. Radiology 261, 274–282. 10.1148/radiol.11110173 21788524 PMC3184233

[B13] Handa T. Tanizawa K. Oguma T. Uozumi R. Watanabe K. Tanabe N.. (2021). Novel artificial intelligence-based technology for chest computed tomography analysis of idiopathic pulmonary fibrosis. Ann. Am. Thorac. Soc. 10.1513/AnnalsATS.202101-044OC. [Epub ahead of print]. 34410886

[B14] Haruna A. Muro S. Nakano Y. Ohara T. Hoshino Y. Ogawa E.. (2010). CT scan findings of emphysema predict mortality in COPD. Chest 138, 635–640. 10.1378/chest.09-2836 20382712

[B15] Kaji S. Kida S. (2019). Overview of image-to-image translation by use of deep neural networks: denoising, super-resolution, modality conversion, and reconstruction in medical imaging. Radiol. Phys. Technol. 12, 235–248. 10.1007/s12194-019-00520-y 31222562

[B16] Kim G. B. Jung K. H. Lee Y. Kim H. J. Kim N. Jun S.. (2018). Comparison of shallow and deep learning methods on classifying the regional pattern of diffuse lung disease. J Digit Imaging 31, 415–424. 10.1007/s10278-017-0028-9 29043528 PMC6113148

[B17] Labaki W. W. Martinez C. H. Martinez F. J. Galban C. J. Ross B. D. Washko G. R.. (2017). The role of chest computed tomography in the evaluation and management of the patient with chronic obstructive pulmonary disease. Am. J. Respir. Crit. Care Med. 196, 1372–1379. 10.1164/rccm.201703-0451PP 28661698 PMC5736976

[B18] Lee C. W. Seo J. B. Lee Y. Chae E. J. Kim N. Lee H. J.. (2012). A pilot trial on pulmonary emphysema quantification and perfusion mapping in a single-step using contrast-enhanced dual-energy computed tomography. Invest. Radiol. 47, 92–97. 10.1097/RLI.0b013e318228359a 21750465

[B19] Lee S. M. Lee J. G. Lee G. Choe J. Do K. H. Kim N.. (2019). CT image conversion among different reconstruction kernels without a sinogram by using a convolutional neural network. Korean J. Radiol. 20, 295–303. 10.3348/kjr.2018.0249 30672169 PMC6342751

[B20] Mcdonald M. L. Diaz A. A. Ross J. C. San Jose Estepar R. Zhou L. Regan E. A.. (2014). Quantitative computed tomography measures of pectoralis muscle area and disease severity in chronic obstructive pulmonary disease. A cross-sectional study. Ann. Am. Thorac. Soc. 11, 326–334. 10.1513/AnnalsATS.201307-229OC 24558953 PMC4028743

[B21] Nishimura M. Makita H. Nagai K. Konno S. Nasuhara Y. Hasegawa M.. (2012). Annual change in pulmonary function and clinical phenotype in chronic obstructive pulmonary disease. Am. J. Respir. Crit. Care Med. 185, 44–52. 10.1164/rccm.201106-0992OC 22016444

[B22] Ohara T. Hirai T. Muro S. Haruna A. Terada K. Kinose D.. (2008). Relationship between pulmonary emphysema and osteoporosis assessed by CT in patients with COPD. Chest 134, 1244–1249. 10.1378/chest.07-3054 18641115

[B23] Otrakji A. Digumarthy S. R. Lo Gullo R. Flores E. J. Shepard J. A. Kalra M. K. (2016). Dual-energy CT: spectrum of thoracic abnormalities. Radiographics 36, 38–52. 10.1148/rg.2016150081 26761530

[B24] Pishgar F. Shabani M. Quinaglia A. C. S. T. Bluemke D. A. Budoff M. Barr R. G.. (2021). Quantitative analysis of adipose depots by using chest CT and associations with all-cause mortality in chronic obstructive pulmonary disease: longitudinal analysis from MESArthritis ancillary study. Radiology 299, 703–711. 10.1148/radiol.2021203959 33825508 PMC8165946

[B25] Popuri K. Cobzas D. Esfandiari N. Baracos V. Jagersand M. (2016). Body composition assessment in axial ct images using fem-based automatic segmentation of skeletal muscle. IEEE Trans. Med. Imaging 35, 512–520. 10.1109/TMI.2015.2479252 26415164

[B26] R Core Team (2021). R: A Language and Environment for Statistical Computing. Vienna: R Foundation for Statistical Computing. Available online at: https://www.R-project.org/ (accessed June 1, 2021).

[B27] Rao C. R.. (1948). The utilization of multiple measurements in problems of biological classification. J. R. Stat. Soc. 10, 159–193. 10.1111/j.2517-6161.1948.tb00008.x

[B28] Ronneberger O. Fischer P. Brox T. (2015). U-Net: Convolutional Networks for Biomedical Image Segmentation. Available online at: https://ui.adsabs.harvard.edu/abs/2015arXiv150504597R (accessed May 01, 2015). 10.1007/978-3-319-24574-4_28

[B29] Tanabe N. Kaji S. Sato S. Yokoyama T. Oguma T. Tanizawa K.. (2021a). A homological approach to a mathematical definition of pulmonary fibrosis and emphysema on computed tomography. J. Appl. Physiol. 131, 601–612. 10.1152/japplphysiol.00150.2021 34138650

[B30] Tanabe N. Sato S. Tanimura K. Oguma T. Sato A. Muro S.. (2021b). Associations of CT evaluations of antigravity muscles, emphysema and airway disease with longitudinal outcomes in patients with COPD. Thorax 76, 295–297. 10.1136/thoraxjnl-2020-215085 32868293

[B31] Tanabe N. Shimizu K. Terada K. Sato S. Suzuki M. Shima H.. (2021c). Central airway and peripheral lung structures in airway disease-dominant COPD. ERJ Open Res. 7:00672-2020. 10.1183/23120541.00672-2020 33778061 PMC7983277

[B32] Tanimura K. Sato S. Fuseya Y. Hasegawa K. Uemasu K. Sato A.. (2016). Quantitative assessment of erector spinae muscles in patients with chronic obstructive pulmonary disease. novel chest computed tomography-derived index for prognosis. Ann. Am. Thorac. Soc. 13, 334–341. 10.1513/AnnalsATS.201507-446OC 26700501

[B33] Vestbo J. Edwards L. D. Scanlon P. D. Yates J. C. Agusti A. Bakke P.. (2011). Changes in forced expiratory volume in 1 second over time in COPD. N. Engl. J. Med. 365, 1184–1192. 10.1056/NEJMoa1105482 21991892

[B34] Vieira S. R. Puybasset L. Richecoeur J. Lu Q. Cluzel P. Gusman P. B.. (1998). A lung computed tomographic assessment of positive end-expiratory pressure-induced lung overdistension. Am. J. Respir. Crit. Care. Med. 158, 1571–1577. 10.1164/ajrccm.158.5.9802101 9817710

[B35] Vogelmeier C. F. Criner G. J. Martinez F. J. Anzueto A. Barnes P. J. Bourbeau J.. (2017). Global strategy for the diagnosis, management, and prevention of chronic obstructive lung disease 2017 report: GOLD executive summary. Eur. Respir. J. 49, 557–582. 10.1183/13993003.00214-2017 28182564

[B36] Williams M. C. Murchison J. T. Edwards L. D. Agusti A. Bakke P. Calverley P. M.. (2014). Coronary artery calcification is increased in patients with COPD and associated with increased morbidity and mortality. Thorax 69, 718–723. 10.1136/thoraxjnl-2012-203151 24473329

[B37] Yamada Y. Jinzaki M. Okamura T. Yamada M. Tanami Y. Abe T.. (2014). Feasibility of coronary artery calcium scoring on virtual unenhanced images derived from single-source fast kVp-switching dual-energy coronary CT angiography. J. Cardiovasc. Comput. Tomogr. 8, 391–400. 10.1016/j.jcct.2014.08.005 25301045

